# Closing the Gap for Children with OCD: A Staged-Care Model of Cognitive Behavioural Therapy with Exposure and Response Prevention

**DOI:** 10.1007/s10567-023-00439-2

**Published:** 2023-07-05

**Authors:** Lara J. Farrell, Allison M. Waters, Eric A. Storch, Gabrielle Simcock, Iain E. Perkes, Jessica R. Grisham, Katelyn M. Dyason, Thomas H. Ollendick

**Affiliations:** 1 School of Applied Psychology & Griffith University Centre for Mental Health, Griffith University, Gold Coast Campus, Southport, QLD 4222 Australia; 2 School of Applied Psychology & Griffith University Centre for Mental Health, Griffith University, Mount Gravatt Campus, Mount Gravatt, Australia; 3 Baylor College of Medicine, Houston, USA; 4 Department of Psychological Medicine, Sydney Children’s Hospitals Network, Westmead, NSW Australia; 5 Discipline of Psychiatry and Mental Health, School of Clinical Medicine, Faculty of Medicine and Health, University of New South Wales, Sydney, NSW Australia; 6 Discipline of Paediatrics and Child Health, School of Clinical Medicine, Faculty of Medicine and Health, University of New South Wales, Sydney, NSW Australia; 7 School of Psychology, University of New South Wales, Sydney, Australia; 8 Child Study Centre, Virginia Polytechnic University, Blacksburg, USA

**Keywords:** OCD, Youth, Children, CBT, ERP, Staged care

## Abstract

Childhood obsessive–compulsive disorder (OCD) is among the most prevalent and disabling mental health conditions affecting children and adolescents. Although the distress and burden associated with childhood OCD are well documented and empirically supported treatments are available, there remains an unacceptable “treatment gap” and “quality gap” in the provision of services for youth suffering from OCD. The treatment gap represents the large number of children who never receive mental health services for OCD, while the quality gap refers to the children and young people who do access services, but do not receive evidence-based, cognitive behavioural therapy with exposure and response prevention (CBT-ERP). We propose a novel staged-care model of CBT-ERP that aims to improve the treatment access to high-quality CBT-ERP, as well as enhance the treatment outcomes for youth. In staged care, patients receive hierarchically arranged service packages that vary according to the intensity, duration, and mix of treatment options, with provision of care from prevention, early intervention, through to first and second-line treatments. Based on a comprehensive review of the literature on treatment outcomes and predictors of treatments response, we propose a preliminary staging algorithm to determine the level of clinical care, informed by three key determinants: severity of illness, comorbidity, and prior treatment history. The proposed clinical staging model for paediatric OCD prioritises high-quality care for children at all stages and levels of illness, utilising empirically supported CBT-ERP, across multiple modalities, combined with evidence-informed, clinical decision-making heuristics. While informed by evidence, the proposed staging model requires empirical validation before it is ready for prime time.

Obsessive–compulsive disorder (OCD) is one of the most prevalent and disabling mental health conditions affecting children and young people (CYP). Lifetime prevalence rates from around the world are estimated to be between 2 and 3% in developed countries (Kessler et al., 2011; Ruscio et al., 2010) and between two-thirds to three-quarters of all adults diagnosed with OCD experience onset by 6 to 12 years of age (Geller et al., 2012; Taylor et al., 2011). Childhood onset OCD is associated with greater OCD severity, more OC symptoms, higher comorbidity, and greater prevalence of OCD in first-degree relatives and has a poorer prognosis and treatment response relative to adult onset OCD (Geller, 2006; Taylor et al., 2011). Despite the well-documented distress and burden associated with childhood OCD (Amir et al., 2000; Piacentini et al., 2003; Wu et al., 2018) and the availability of empirically supported treatments (McGuire et al., 2015), there remains an unacceptable “treatment gap” and “quality gap” in the provision of services for CYP suffering from OCD. The “treatment gap” represents the difference between the 1 in 50 children who suffer from OCD at any point in time, and the negligible proportion who ever receive evidence-based treatment. The gap is further evident in the staggering delays in time to care, with Australian data suggesting more than 9 years of untreated illness for OCD (Cooper et al., 2023). Moreover, even when families do access services, they rarely receive evidence-based care, and if they do, often it is delivered sub-optimally (), thereby highlighting the “quality gap” in available services.

Cognitive-behavioural therapy, with a focus on exposure and response prevention (CBT-ERP), is highly effective for CYP with OCD (McGuire et al., 2015) with almost two decades of empirical support since the first published randomised controlled trials (Barrett et al., 2004; POTS, 2004). However, CBT-ERP continues to be underutilised in community settings and infrequently implemented with only ~ 5% of therapist *routinely* delivering the gold-standard, therapist-delivered, within-session CBT-ERP (). COVID-19 has also disproportionately and profoundly impacted younger people, and especially young people with pre-existing mental health conditions such as OCD. Indeed, there is mounting evidence of worsening of OCD symptoms among youth already affected since the pandemic (Cunning & Hodes, 2022; Van Ameringen et al., 2022), as well as rising rates of OCD among youth more generally, and especially those with other pre-existing mental health problems (Kroon et al., 2022). This staggering increase in mental illness generally, and OCD in particular, has coincided with severe disruptions to mental health services, a sector already under enormous pressure, resulting in an unacceptable gap in the provision of high-quality psychological services.

In recent years, stepped care models have been proposed as one solution to improve the access to care. Stepped care involves the delivery of treatments from the least restrictive (in time/costs/specialisation: e.g. self-help; digital mental health with minimal therapist assistance) to the most intensive, whereby patients are only “stepped up” when they fail at an earlier step (Ollendick et al., 2018). Stepped care has been found to be effective for child anxiety disorders (Ollendick et al., 2018; Rapee et al., 2017); however, it may be less suited to severe conditions such as OCD, where unnecessary delays in adequate dosing of ERP may result in higher illness-related burden and costs (Diefenbach & Tolin, 2013). More recently, staged-care models for youth mental health service provision have been proposed (Hickie et al., 2019) and are informed by the medical concept that less advanced stages of disease require less intensive treatment, compared to advanced stages of disease that require more intensive treatment (Hickie et al., 2019). Staged care therefore personalises treatment selection for individuals based on the clinical severity of the presenting problem, the individual's illness trajectory, understanding of underlying mechanisms and current comorbidities to inform the clinical stage (Cross et al., 2019), thereby delivering “right care, first time” (Hickie et al., 2019). This review paper aims to outline the ongoing treatment and quality gaps that exist for CYP with OCD and proposes a novel approach to improve the high-quality care through access to evidence-informed treatments that are staged, based on the clinical characteristics of CYP with OCD. The staged-care model of CBT-ERP presented here is informed by more than two decades of empirical research evaluating CBT-ERP for CYP with OCD, delivered across multiple modalities, and examining the clinical predictors of treatment outcomes to inform clinical decision-making that determines level of care for CYP with OCD at all stages of illness progression.

## Impact of OCD on Children and Young People

The onset of OCD in childhood or adolescence can disrupt the critical development that takes place throughout these important stages of life (Fineberg et al., 2019) and often negatively impacts social, family, and academic functioning (Cooper, 1996; Piacentini et al., 2003; Storch et al., 2010; Valderhaug & Ivarsson, 2005). In many cases, uncontrollable distress, avoidance of obsessional triggers, and time-consuming rituals interfere with the child’s routine and lead to difficulties with school attendance and difficulty interacting with peers (Farrell & Barrett, 2007). Up to 90% of children with OCD endorse significant functional impairment across multiple domains of life (Lack et al., 2009; Piacentini et al., 2003; Valderhaug & Ivarsson, 2005) and, moreover, report significantly lower quality of life relative to community controls (Coluccia et al., 2017). Childhood OCD symptoms and the associated impairment often persist into adulthood if not treated early (Freeman et al., 2014).

The negative effects of OCD are not limited to the personal experience of young people, but extend to their home life and family functioning as well (Stewart et al., 2017). OCD is directly associated with increased family distress (Albert et al., 2010; Amir et al., 2000) and indirectly linked to reduced family functioning and organisation (Calvocoressi et al., 1995; Maina et al., 2006). Indeed, the emotional burden of OCD on family members has been found to be similar or even greater than the family burden associated with mood (Vikas et al., 2011) and psychotic disorders (Remmerswaal et al., 2019). The impact of OCD on the family is further exacerbated when family members become directly involved in the disorder via accommodation behaviours (Lebowitz, 2016), which the caregiver performs to assist individuals with OCD to avoid or manage their anxiety (Lebowitz et al., 2012). A child with OCD may ask family members to provide repeated reassurance, participate in compulsions, help avoid obsessional triggers, or delay daily activities to complete elaborate rituals (Abramowitz et al., 2013). Indeed, over 96% of relatives of individuals with OCD report engaging in accommodation behaviours (Lebowitz et al., 2012), and nearly half of mothers and one third of fathers report daily occupational impairment as a result of managing child OCD symptoms and family accommodation (Stewart et al., 2017).

Despite being well intentioned, family accommodation worsens OCD symptoms, negatively impacts treatment response, and contributes to higher levels of functional impairment in children and adolescents (Peris et al., 2008; Storch et al., 2007). Accommodation of OCD also has direct repercussions for family members, depleting emotional resources, reducing attention available for other family members, and altering the family’s schedule (Lebowitz, 2016; Lebowitz et al., 2012). OCD frequently also severely disrupt family life due to the expressed emotion of the affected individual when family members refuse to accommodate (Stewart et al., 2017). Early detection and delivery of efficient and effective treatment are critical to offset the profound impairment, disability, and economic burden of paediatric OCD. Moreover, interventions that support families and parents in managing OCD symptoms at home, reducing family accommodation, and supporting implementation of ERP are necessary for high-quality, effective care.

## Evidence-Based Treatment for CYP with OCD

The two most widely supported treatments for paediatric OCD are cognitive-behavioural therapy with exposure and response prevention (CBT-ERP) and serotonin-reuptake inhibitors (SRIs)—either alone or in combination (Geller et al., 2012; McGuire et al., 2015). CBT-ERP is the most widely supported evidence-based psychosocial treatment for CYP with OCD and current clinical guidelines recommend CBT-ERP treatment as the first-line intervention, with the possible addition of SRI medication for those with moderate or severe OCD, or those with limited response to CBT-ERP (Geller et al., 2012; McGuire et al., 2015).

### Cognitive-Behavioural Therapy with exposure and response prevention (CBT-ERP)

CBT is a psychological treatment that involves both challenging thoughts (cognitive) and changing behavioural patterns (behavioural). ERP involves exposing CYP with OCD to situations or stimuli that trigger obsessions (exposure) and distress, while they are encouraged to resist the urge to complete compulsions (response prevention; Wu et al., 2016). In doing so, individuals learn new associations with feared stimuli, and new coping strategies for managing distress, thus breaking the cycle of OCD (Craske et al., 2008; Himle & Franklin, 2009).

Several meta-analyses comprising hundreds of children and adolescents have found CBT-ERP to be highly effective at reducing symptoms of OCD (Ale et al., 2015; McGuire et al., 2015; Öst et al., 2016; Reid et al., 2021; Skapinakis et al., 2016; Uhre et al., 2020; Wu et al., 2016); although Uhre et al (2020) conclueded that risk of bias across randomised controlled trials (RCTs) to date is high, with blinding of assessors and concealment of allocation not always reported or achieved in psychotherapy trials. The most recent meta-analysis, however, including CYP (*n* = 537) and adults (*n* = 1483) with OCD demonstrated that CBT-ERP was associated with a large effect size (*g* = 1.09) for CYP compared to a variety of other treatments (psychological, pharmacological, placebo) or waitlists (Reid et al., 2021). Moreover, CBT-ERP for CYP was found to be as, or more, effective than CBT-ERP for adults (*g* = 0.60), with effectiveness of CBT-ERP significantly reducing with age (*p* = 0.006). In clinical trials, approximately 70% of CYP achieve treatment response (i.e. clinically significant reduction in symptoms) and 60% full remission (i.e. no longer meeting criteria for OCD (McGuire et al., 2015). These gains are maintained after treatment, for at least 6 to 9 months (Franklin et al., 2015; Melin et al., 2020). Further, empirical research shows that ERP is typically tolerable with minimal side effects and low attrition (McGuire et al., 2018; Piacentini et al., 2007), acceptable to patients (Skapinakis et al., 2016), and CBT/ERP with or without the addition of an SSRI is the most cost-effective treatment option for children and adolescents (Skapinakis et al., 2016).

#### Modes of Delivery

Typically, CBT-ERP is delivered for 12 to 20 sessions (Gryczkowski & Whiteside, 2014) and can be delivered in a variety of formats (individual, group, family) while maintaining effectiveness (Barrett et al., 2004; Öst et al., 2016). Group-based treatment may offer an economy of scale, meaning more clients can receive treatment from one or two clinicians (Himle et al., 2003), and for OCD, group therapy also offers peer support, reducing the secretive nature of OCD and normalising the symptoms (Lavell et al., 2016). Group therapy may also increase compliance with ERP and motivation for change, via within-group norms and modelling processes (Lavell et al., 2016). Family-based treatment addresses family systems that children operate within and can incorporate parent management training or behaviour management techniques (Franklin et al., 2015). In the treatment of OCD with CYP, family involvement is considered an essential part of effective CBT-ERP, given that family accommodation and associated distress in family members is rife and is associated with attenuated outcomes (Thompson-Hollands et al., 2014).

In the only large-scale effectiveness study of CBT-ERP (*n* = 269, aged 7–17) delivered within community mental health clinics (the Nordic Long-Term OCD Treatment Study, NordLOTS), Torp et al. (2015) examined the outcomes for CYP with OCD following 14 sessions of manualised CBT-ERP delivered across 20 clinics in Norway, Denmark, and Sweden. Outcomes demonstrated that treatment engagement/adherence was high (nine out of 10 patients completed the full protocol) and on average participants’ OCD symptoms reduced by 52.9% following CBT, with a large, estimated effect size of 1.58. This study provides support for the implementation of CBT-ERP in community routine mental health services, using evidence-based manuals, supported by high-quality clinician training (i.e. up to 10 days clinician training) and site-specific supervision to support implementation in routine care (i.e. 3-hourly monthly site supervision).

### Predictors and Moderators of CBT-ERP Treatment Response

Although CBT-ERP is considered the gold-standard first-line treatment for childhood OCD (Torp et al., 2015) not all patients achieve complete remission from CBT-ERP for OCD despite reduction in symptoms (Öst et al., 2016). For example, in the POTS (2004) RCT, 46% of CYP receiving combined CBT and serotonergic reuptake inhibitor (SRI) medication did not fully remit from OCD, whereas 60% of those receiving CBT alone, and 80% of those receiving SRI alone did not achieve complete remission; highlighting significant room for improvement in treatment outcomes. Given this, interest has turned to exploring the predictors and moderators of OCD treatment response which can help inform which children will respond best to CBT-ERP, and who will require more refined, personalised approaches. McGuire et al. (2015) in their meta-analysis (*n* = 20 studies) found that a greater number of comorbid anxiety disorders, and a greater number of therapeutic contact hours was associated with stronger CBT efficacy and symptom response, while greater treatment attrition was associated with lower treatment efficacy. Keeley et al. (2008) in their early review of the literature examining the predictors of treatment response concluded that whist there is variable outcomes across studies, the most consistent predictors of poorer response to CBT included increased OCD symptom severity, more severe comorbid depression, greater family dysfunction, and reduced therapeutic alliance.

Garcia et al. (2010) examined the moderators of response based on the POTS RCT (2004) and identified that family history of OCD moderated treatment response for CBT, but not other conditions. Youth with a family history of OCD who received CBT alone had a more than sixfold decrease in effect size (ES = 0.25) relative to those without a family history of OCD (ES = 1.63). The authors concluded that a family history of OCD may attenuate CBT response specifically, given that CBT generally requires substantive family support and engagement (e.g. with ERP homework), relative to medication compliance.

#### OCD Severity and Comorbidity

OCD severity at pre-treatment has often been found to be a significant predictor of treatment outcome (Garcia et al., 2010; Ginsburg et al., 2008), although not all studies have found this association (e.g. Torp et al., 2015). A recent study (*N* = 314, youth aged 13–17 years) demonstrated that lower baseline OCD severity was the most robust predictor of lower post-treatment OCD severity and that other predictors expected to be associated with post-treatment OCD severity that were entered into the model were not significant, including length of treatment and comorbid symptoms of anxiety and depression (Højgaard et al., 2020) Piacentini et al. (2002) similarly found that higher baseline OCD severity and OCD-related impairment were the only significant predictors of poorer treatment response. Other studies, however, have found that in addition to baseline OCD severity, mental health comorbidities are also associated with OCD treatment outcomes.

In a large study, examining 15 possible predictor variables from the Paediatric Obsessive Compulsive Treatment Study (POTS, 2004), Garcia et al. (2010) identified only five significant variables associated with poorer treatment outcomes and response, including baseline OCD severity, impairment, insight, comorbid externalising symptoms, and family accommodation (Garcia et al., 2010). Indeed, several other studies have identified comorbid disruptive behaviour disorders, ADD/ADHD, and depression (but not anxiety), as associated with negative treatment outcomes (Farrell et al., 2012a, 2012b; Storch et al., 2008). Farrell et al., (2012a, 2012b) for instance examined the impact of specific comorbid presentations (i.e. ADHD, autism spectrum disorder, depression) on treatment response and remission rates in children and youth following group CBT. While they did not find a significant predictive relationship immediately post-treatment, treatment outcomes were attenuated at 6-month follow-up for youth with multiple comorbid conditions, and for those with ADD/ADHD, but not other comorbid conditions.

Jassi et al. (2021) recently reported evidence from a large clinical sample that youth with comorbid OCD and ASD (*n* = 100) had poorer treatment response to CBT-ERP for OCD, relative to youth without ASD (OCD sample, *n* = 223). These findings are consistent with those reported by Griffiths et al. (2017) who found CYP with comorbid OCD and ASD (*n* = 25) experienced significantly poorer treatment response at six-month follow-up relative to CYP with OCD and no comorbid ASD (*n* = 25). There is recent evidence with adults that diminished working memory and poor communication skills, which may underlie comorbid presentations of ADD/ADHD and ASD, are associated with poor responsiveness to CBT-ERP for OCD. However, Hybel et al. (2017) found that higher baseline executive functioning reduced the effectiveness of CBT on treatment outcomes in CYP, in contrast to children with lower baseline executive function scores. The finding that comorbid depression (severe), ADHD, and ASD appear to be associated with an attenuated response to CBT-ERP for OCD, but not comorbid anxiety symptoms / disorders, might be because those conditions are more clearly distinct from OCD (i.e. there may be diagnostic over-shadowing of OCD with co-occurring anxiety for instance), and (arguably) more often require management with adjunctive pharmacological intervention. Furthermore, the functional difficulties experienced with ADHD/ASD may constitute a continuous burden in CYP lives, which may also serve to maintain OCD symptoms.

#### Age and Length of Illness

There are almost no studies that have found age, or duration of illness as significant predictors of CBT-ERP outcomes in CYP with OCD. In a meta-analysis of 16 RCTs across the lifespan (*n* = 756 patients), Olatunji et al. (2013) found no moderating effects of age on outcomes within child or adult samples; however, they did report more favourable outcomes for CYP relative to adult samples. They found no evidence for duration of illness or age of onset as predictors of outcome. However, the NordLOTS study of CBT-ERP in community mental health clinics (*N* = 269) for CYP with OCD examined 20 potential predictor variables for responsiveness to CBT-ERP and found that in the multivariate model, only younger age at treatment was a significant predictor of more favourable CBT outcomes, and that younger children (aged 7–11 years) generally experienced lower OCD severity relative to adolescents (aged 12–17 years) at post-treatment. Notably, in their model with age, OCD duration and their interaction were not significant, with only age (younger versus older) a significant predictor of more favourable outcomes to first-line CBT-ERP in community clinics (Torp et al., 2015).

#### Family Functioning

Family factors are also associated with OCD in CYP and treatment outcomes. For example, family dysfunction (Barrett et al., 2005), family history of OCD (Garcia et al., 2010), and parental psychopathology (Leonard et al., 1993) can predict poorer treatment outcomes for youth with OCD. Additionally, parenting styles that are characterised by parental rejection at baseline are associated with higher OCD symptoms at post-treatment and children of families who report high levels of family accommodation to OCD-related behaviours have higher OCD symptom severity at 12 months post-treatment (Lavell et al., 2016). Moreover, family accommodation is linked with greater OCD severity (Lebowitz et al., 2012) and lower functionality (Caporino et al., 2012). It also predicts treatment outcomes in that higher pre-treatment rates of family accommodation are associated with poorer treatment outcomes (Gorenstein et al., 2015) and that greater improvements are made with lower levels of family accommodation (Garcia et al., 2010; Peris et al., 2017).

A recent meta-analysis (McGrath & Abbott, 2019) explored the moderating role of family-related treatment variables, including family accommodation, blame, family cohesion and conflict, and general family functioning for youth with OCD. Data from 37 eligible studies (1727 youth, mean age range of 5.8 to 14.5 years) demonstrated that family-based interventions were effective in reducing youth OCD symptoms and family accommodation at post-test and follow-up, with large treatment effect sizes. Moreover, targeting a greater number of family factors was associated with greater reductions in family accommodation from pre-treatment to post-treatment. Surprisingly, however, the greater the number of family factors targeted in treatment did not significantly moderate OCD symptom severity (C/Y-BOCS) at post-treatment, perhaps due to the small number of studies that assessed family accommodation at pre-treatment to post-treatment. This meta-analysis demonstrates the importance of addressing a range of family factors in youth OCD treatment to optimise the outcomes.

## The Treatment Gap and Quality Gap for CYP with OCD

Few patients with OCD ever receive gold-standard CBT-ERP, with adult estimates suggesting 60% of patients do not receive *any* treatment for OCD (Kohn et al., 2004). In Australia, results from a national survey of over 6,300 families with children aged 4 to 17 years, indicated that only ~ 5% of CYP with mental health disorders accessed specialist child and youth mental health services in the 12 months prior to the survey (Lawrence et al., 2016). Furthermore, in a recent study of adults with OCD and/or caregivers of those with OCD, the mean time of untreated illness with OCD was 9 years (Cooper et al., 2023). Even when families do access services, rarely do they receive evidence-based care (i.e. CBT for OCD and anxiety), and if they do, often it is delivered sub-optimally (Deacon & Farrell, 2013; Deacon et al., 2013; Reid et al., 2018). Indeed, children with OCD are unlikely to receive gold-standard CBT-ERP outside of specialist centres, with one study in the US reporting the majority of youth (> 70%) with anxiety disorders presenting to non-specialist centres are never introduced to exposure therapy during treatment (Reid et al., 2018; Whiteside et al., 2016a). Numerous barriers to accessing CBT-ERP have been highlighted, including a lack of trained clinicians, geographical and financial barriers, limited access to services, and the time-intensive nature of traditional weekly therapy (Goisman et al., 1993; Marques et al., 2010b; Turner et al., 2009). Geographical constraints are of course an ongoing major problem for families residing outside of metropolitan areas. Youth with OCD residing in regional areas face problems with healthcare workforce shortages, lack of local clinician’s trained in CBT-ERP protocols, and time and transportation costs if travelling long distances to urban areas for treatment (Comer et al., 2014; Crum & Comer, 2016). Even in metropolitan areas, there remains a dearth of qualified, trained clinicians who can competently deliver CBT-ERP for paediatric OCD. Moreover, clinician and patient negative perceptions about CBT-ERP present another major barrier to implementation and access in routine care (e.g. reluctance to engage in exposure therapy; Young et al., 2014).

### Geographical, Structural, and Organisational Factors

The logistics and structural factors associated with delivering ERP have been identified as a major barrier to therapeutic implementation. Session length and access to resources needed, such as off-site locations or stimulus materials, to implement exposure therapy were among the primary reasons that clinicians reported not utilising ERP in practice (Reid et al., 2017). A survey of members of the Dutch Association for CBT (*n* = 490) found that 55.3% of the respondents were not satisfied with the exposure resources at their workplace in terms of lack of proper protocols, while 22.2% also reported an insufficient availability of materials supporting the implementation of exposure (Sars & van Minnen, 2015). Furthermore, evidence-based treatment manuals for OCD recommend concentrated exposure sessions ranging from 90 to 120 min (Lewin et al., 2005), which is considerably longer than standard sessions (i.e. 50 min) and therefore may not be covered by government rebates or insurance companies (Reid et al., 2017). Indeed, Marques et al. (2010a) survey of 175 people with OCD reported that one of the main reasons they did not receive exposure treatment was due to the high cost of therapy and lack of insurance coverage accepted by providers.

### Clinician Factors

Due to clinicians’ concerns about safety and tolerability of exposure therapy, CBT-ERP is often underutilised in community clinics. Many clinicians believe, for example, that exposure therapy is aversive to clients (Rothbaum & Schwartz, 2002), may exacerbate symptoms (Olatunji et al., 2009), cause harm (Richard & Gloster, 2007), or lead to treatment dropout (van Minnen et al., 2010). Even when ERP is delivered, it is often implemented in an overly cautious manner that may limit its effectiveness (Farrell et al., 2016). For example, clinicians may avoid using exposure tasks that cause high anxiety in the client or they may terminate the exposure tasks prematurely (Deacon & Farrell, 2013).

A survey of 182 clinicians examining their likelihood of excluding clients from ERP revealed that clinicians with increased anxiety and sensitivity and who held negative beliefs about exposure therapy were less likely to utilise the treatment method (Meyer et al., 2014) Another recent survey of 107 clinicians who treat youth with OCD showed that varying levels of exposure therapy were utilised depending on clinician background: clinical psychologists with a CBT orientation were more likely to implement ERP, compared to other allied health workers with differing treatment preferences. Moreover, therapists who more often utilised ERP with CYP with OCD had more positive attitudes to exposure therapy (Keleher et al., 2020).

Specific to utilising exposure therapy with youth with OCD, Reid et al. (2017) found that among the top reported barriers to treatment were clinician’s (*N* = 230) concern about parents reacting negatively to ERP or believing that young people *are not developmentally equipped to handle the treatment.* Characteristics of OCD may also be a barrier to treatment. For example, in Keleher et al. (2020) study, clinicians reported that they were less likely to use ERP on OCD sub-types of hoarding or taboo obsessions, compared to contamination or cleaning rituals, symmetry obsessions or ordering compulsions, and mental rituals. Further, in a survey of 216 German clinicians (Moritz et al., 2019) nearly half (49.1%) the clinicians reported that they were unable to implement exposure therapy as the client refused the treatment. The clinicians were also less likely to implement exposure therapy if the client lacked motivation to engage in the therapeutic process or if perceived somatic constraints prohibited the procedure. In sum, these unfounded negative perceptions and concerns about the effects of ERP reduce the likelihood of clinicians implementing this gold-standard treatment, thereby creating a treatment and quality gap for youth with OCD.

### Sub-clinical OCD

In addition to the 2 to 3% of CYP with clinical levels of OCD, there is a larger proportion of CYP with sub-clinical symptoms, or emerging OCD, that to date very little is known about. Notably, however, these CYP with sub-clinical OC symptoms experience similar levels of distress, impairment, and comorbidity to CYP with a diagnosis of OCD (Fullana et al., 2009), yet rarely receive any form of early intervention. Thus, CYP with sub-clinical OCD also fall into the treatment gap and represent a sub-group of young people with unmet needs. The notion that treatment interventions should also be extended to sub-clinical OCD symptoms has recently been raised in the literature (Fontenelle & Yücel, 2019) and is supported by evidence that sub-clinical OCD during childhood is indeed a robust predictor of full diagnosis in adulthood (Fullana et al., 2009), yet these CYP are typically overlooked for treatment.

To date there is limited research reporting on definitions of sub-clinical OCD or rates at which CYP experience sub-threshold OC symptoms. One Spanish study (Canals et al., 2012), including 562 children aged 9 to 13 years of age, showed that the prevalence of OC diagnosis was 4.75% (CI 95% 3.73–5.95), while the prevalence of sub-clinical OCD was an additional 5.5% (CI 95% 4.3–6.6%). Furthermore, children’s global assessment scores (CGAS) (Shaffer et al., 1983) indicated significantly poorer global functioning for children with sub-clinical OCD (*M*: 72; *SD*: 22) relative to youth with no OCD symptoms (*M*: 93; *SD*: 7), despite being significantly higher than children diagnosed with OCD (*M*: 61; *SD*: 12).

Similar findings of reduced functioning in youth with sub-clinical OCD have also been reported in a Brazilian community sample of 1,554 children aged 6–12 years with either a diagnosis of OCD, sub-clinical OCD (i.e. one or more obsession or compulsion symptoms), or no symptoms (Alvarenga et al., 2015). They found no significant differences between youth with an OCD diagnosis or sub-clinical OCD symptoms in functional impairment, school problems, delinquent behaviour, and treatment-seeking behaviours; and both groups were significantly more impaired than the healthy controls. Moreover, psychiatric comorbidities between youth with an OCD diagnosis, or youth with OCD symptoms, did not differ and both these groups had significantly higher comorbidities than youth with no OCD symptoms. While there is limited research examining symptom trajectories among CYP with sub-clinical OCD, there is evidence for convergence into clinical OCD later in life.

The Dunedin multidisciplinary prospective longitudinal cohort study assessed OCD symptoms among children at 11 years of age (*n* = 792) and followed the cohort up at ages 26 years, and 32 years (Fullana et al., 2009). They found that youth experiencing one or more obsessive or compulsive symptoms (8% of the sample) were significantly more likely to be diagnosed with OCD as adults (odds ratio 5.90, 95% CI 2.18–15.9). Further, 11-year-olds with OCD symptoms were significantly more likely to experience symptom dimensions of contamination/cleaning (odds ratio 3.37, 95% CI 1.32–8.61) and shameful thoughts (odds ratio 2.38, 95% CI 1.17–4.81) in adulthood. Given that early onset sub-clinical OCD is prevalent in the community, is associated with high functional impairment and other psychiatric comorbidities, and represents a gateway to full blown diagnosis of OCD in adulthood (Fullana et al., 2009) there is compelling reasons to screen, detect and treat CYP with sub-clinical symptoms of OCD.

## Closing the Treatment and Quality Gap: Innovations in CBT-ERP for CYP with OCD

In recent years, CBT has been adapted and trialled in several different modalities in efforts to improve access to care via more efficient treatment delivery. Innovations include internet-delivered CBT (iCBT: 12 + web-based sessions, (Aspvall et al., 2020; Lenhard et al., 2017); or web-cam / videoconference CBT (Storch et al., 2011); and brief, intensive CBT-ERP delivered in concentrated format over several days, instead of weeks (e.g. 2–3 sessions, (Farrell et al., 2016, 2022); 5 days (Canavera et al., 2022; Whiteside et al., 2014). Stepped care models have also been proposed more broadly in the literature to address the treatment gap in mental health service provision and are based on the tenet that patients be offered the least restrictive treatment (i.e. low intensity intervention) in the first instance, and then stepped up to a higher intensity intervention, as needed if there is an inadequate clinical response at earlier steps (e.g. Ollendick et al., 2018; Salloum et al., 2022).

### Digital Innovations

New technology and increased access to the internet have resulted in a burgeoning field of research aimed at improving access to CBT via digital technology. The most widely studied is that of internet-delivered CBT (iCBT), which ranges from fully automated and self-directed web-based CBT, or some version of therapist-assisted delivery of web-based CBT to videoconference, real-time, therapist-delivered CBT over the internet. A recent systematic review of iCBT for CYP with OCD found only 6 studies (*I* = 96; (Babiano-Espinosa et al., 2019) including three randomised controlled trials (RCTs), one single-case multiple-baseline design, one open-label trial, and one case series. Of these studies, 5 of 6 reported significant decreases in OCD severity ratings from pre- to post-treatment, and the case-study similarly reported symptom reduction for all CYP. The reduction in OCD symptoms ranged from 26% in web-based therapist-assisted iCBT (Lenhard et al., 2017) to 56% reduction in videoconference, real-time therapist-delivered CBT (Storch et al., 2011).

The only RCT of web-based, therapist-assisted CBT for OCD in CYP, involved 67 youth aged 12 to 17 years who were randomised to either 12-week iCBT including therapist and parent support or a waitlist control condition (Lenhard et al., 2017). CYP showed significant improvement in symptoms relative to the waitlist condition (i.e. 26% reduction in symptoms) and were satisfied with the intervention and maintained gains to the 3-month follow-up. In a longer-term follow-up, there were further improvements in OCD symptoms observed from 3 month follow-up to 12 months follow-up, with remission rates increasing from 18 to 40% of the sample (Lenhard et al., 2020). Collectively, the evidence to date suggests it is both feasible and effective to deliver CBT via the internet to increase access to care for CYP with OCD. In particular, iCBT may be particularly effective as a first-line intervention for CYP with OCD within a stepped-care model of service provision (Ollendick et al., 2018), freeing up more intensive interventions for those with greater need (Lenhard et al., 2017).

Although iCBT offers much promise, the interventions with greatest reach potential (web-based, minimal therapist support) have demonstrated substantially lower effects than those observed with interventions involving greater therapist support (Babiano-Espinosa et al., 2019). One potential reason for the initial weaker effects observed in web-based iCBT may be the lower dose–response, given that patients infrequently complete a full CBT program of 12 + weekly sessions when delivered online (Hadjistavropoulos et al., 2017) and thus, the dose of CBT-ERP may be too little for some CYP with OCD.

Given that technology and parents are both crucial enablers of earlier access to CBT-ERP for children with OCD, our Australian team (Farrell et al., 2022a) piloted a novel parent training intervention in ERP (FAST CBT: Families Accessing Skills Training in CBT-ERP for OCD) delivered directly to parents of CYP with moderate to severe OCD (*n* = 8) aged 8–14 years. CYP were randomly assigned to a 2-week or 3-week baseline phase followed by the intervention, which consisted of 4 weekly videoconference sessions (1 h each), plus brief telephone maintenance over three weeks. Results indicated significant improvements in OCD severity and significant reductions in family accommodation to OCD, with 64% of CYP deemed responders at 2-month follow-up. This proof-of-concept trial provides preliminary support for a brief, internet-delivered, parent-focussed ERP intervention (~ 5 h therapist time).

Extending on the work above to further improve reach and efficiencies in treatment delivery, our Australian team has since developed a multi-technology version of FAST CBT, that trains parents as ‘ERP coaches’ via four self-directed, web-based modules, complimented by four weekly videoconference group sessions with a therapist, and a final videoconference group booster session one-month later. The aim of the intervention is to equip families with effective therapeutic strategies for OCD (i.e. ERP) using brief interventions that can be readily accessed online, thereby increasing earlier access and more immediate relief to children and families in need. We have conducted a small pilot of the feasibility and acceptability of the intervention among parents of children (aged 7 to 14 years) with a primary diagnosis of OCD (*n* = 4) using a case series design (Scott et al., in prep). Parents completed four weekly, self-directed web-platform modules coaching them in how to implement ERP with CYP with OCD. This was complemented by four therapist-led, videoconference group sessions. Following the intervention, parents continued to deliver CBT-ERP at home and engaged in two brief therapist check-ins via phone (over a three-week period), and one therapist-led group booster session 1 month later.

Results supported the feasibility of the intervention and indicated high rates of acceptability for the parent training program as a blended, multi-technology modality. Overall, the usability of the web-platform was rated by parents as within the “best imaginable” range, with 3 of the 4 parents deemed “promoters” of the intervention, and one parent (who only completed 50% of the modules) as “passive” user. Decreases in OCD severity were observed on parent, child and clinician-rated measure. The outcomes highlight the opportunity for more efficient models of treatment delivery to reduce barriers to access for families experiencing OCD. Indeed, this novel, multi-technology approach offers promise for early intervention/prevention, as well as augmented care for child-focussed treatment.

### Brief and Intensive CBT-ERP

Given the numerous barriers to accessing CBT-ERP, another feasible option is to increase accessibility to empirically supported treatments by implementing treatment in more time-intensive formats. Brief, intensive treatments offer the same treatment components to weekly modalities, but are condensed into daily sessions, often of longer duration, over a much briefer time frame (Ollindick, 2004). Intensive treatments have demonstrated comparable success to traditional CBT for a variety of anxiety disorders (Öst & Ollendick, 2017). Delivery of CBT-ERP in a brief but concentrated format offers several benefits. Massed exposure allows for prolonged and continuous exposures over a shorter duration of treatment, which increases the speed of recovery from traditionally 3 + months of weekly CBT-ERP to a matter of days and weeks in the case of intensive delivery CBT-ERP (Farrell et al., 2016; Lewin et al., 2005; Storch et al., 2007). This consistent focus on managing OCD symptoms during a concentrated period of time may also increase motivation for the child and family to make treatment their primary focus and commitment (Ollindick, 2004).

Massed, prolonged exposures delivered in an intensive format also offers an adjunct or second-line approach for more severe or treatment resistance patients who have not achieved an adequate response from weekly CBT-ERP (Storch et al., 2007). Recently, cumulative research has demonstrated the feasibility, acceptability and effectiveness of brief, intensive and concentrated CBT-ERP for CYP with OCD, delivered in multiple formats, including 14 sessions across 3 weeks (Storch et al., 2007), 10 sessions over 5 days (Canavera et al., 2022; Whiteside et al., 2014), ~ 22 h over 4 days (Riise et al., 2018) or ~ 9 h over 2 to 3 sessions (2022b; Farrell et al., 2016).

The most concentrated and brief of the intensive CBT-ERP modalities to date is that developed by our team, consisting of 2 to 3 (daily or weekly) sessions of three-hour long ERP, combined with brief telehealth maintenance over a further period of three weeks (15-min telehealth sessions). In an initial controlled, multiple-baseline case series (*n* = 10, (Farrell et al., 2016), the preliminary efficacy of the intensive treatment, involving one psychoeducation session, two sessions of 3-h ERP plus telehealth maintenance delivered once per week for 3-weeks (via videoconferencing), was examined across parent–child- and clinician-rated measures at post-treatment and 6-month follow-up. Overall, there were significant reductions in OCD severity, diagnostic severity and improvements in quality of life, with the majority of the sample (80%) considered reliably improved and achieving clinically significant change. At post-treatment, 80% were considered responders, and 60% were in remission of symptoms, the latter increasing to 70% at 6-month follow-up.

In a more recent large RCT of d-Cycloserine augmented intensive ERP (three sessions of 3-h ERP) for CYP with OCD relative to placebo controlled intensive ERP (*n* = 100, children 7 to 17 years), there were significant reductions in OCD severity and diagnostic severity, and improvements in functioning, for youth across both conditions following intensive ERP (Farrell et al., 2022b). At 6-month follow-up, between 69 and 74% of youth were responders (based on most recent and conservative responder criteria (Farhat et al., 2021), and 49% to 51% were in remission following intensive ERP (Farrell et al., 2022b). Thus, there is good empirical support for brief, intensive ERP for CYP with OCD which could improve access to specialised treatment, as well as offer additional dose–response for CYP who might require more than weekly CBT or iCBT.

There are only a few studies which have examined predictors of response to intensive CBT-ERP for paediatric OCD. One study by Rudy et al. (2014) examined the clinical characteristics as predictors of response to intensive CBT-ERP, delivered over three weeks (*n* = 78) and found that only baseline OCD severity and family accommodation predicted remission status, whereas comorbidity was not found to be a significant predictor. The authors note, however, that the treatment was overall very effective, so even youth with severe OCD achieved a good clinical response with an overall response rate of 88.5% in this study. The authors noted that youth with more severe OCD, and those with greater family accommodation would likely benefit from more intensive delivery of CBT-ERP (over days, instead of weeks) and a stronger family-based approach where parents are better supported in addressing family accommodation. Similarly, Højgaard et al., (2020) examined the predictors to intensive residential treatment of OCD among adolescents with treatment-resistant OCD (*n* = 314) and found that while lower OCD severity at baseline predicted lower severity following treatment, there were no significant predictors of treatment response. Collectively, these studies suggest that intensive CBT-ERP may be more robust against clinical characteristics that otherwise attenuate response to weekly CBT-ERP (i.e. comorbidity).

### Stepped Care

In recent years, stepped care models have been proposed to address the treatment gap by harnessing the efficiencies of different modalities. Stepped care involves the delivery of treatments from the least restrictive (in terms of time/costs/specialisation, e.g. iCBT) to the most intensive, whereby patients are only “stepped up” when they fail to achieve a response at an earlier step (Ollendick et al., 2018). The basis for stepped care models is that there is more than one modality (i.e. intensity of care) of treatment available for a specific condition and that the model of care is self-correcting (Bower & Gilbody, 2005) that is clinicians are skilled in effectively monitoring symptoms and flexibly adjusting treatment to the level of clinical need.

Aspvall et al. (2021) have recently published outcomes of internet-delivered CBT-ERP stepped care (16 sessions iCBT Step 1) relative to in-person CBT-ERP (16 sessions) for CYP with moderate severity OCD (mean CY-BOCS = 23) aged 8 to 17 years (*n* = 152), across two specialist mental health services in Sweden. Non-responders to each condition were offered a further course of in-person CBT-ERP (Step 2). Outcomes indicated that at 3-month follow-up, there were 46% non-responders in stepped care, relative to 30% non-responders in face-to-face CBT-ERP; however, at 6 months follow-up, stepped care was found to be non-inferior to in-person CBT-ERP with 68% of CYP across both conditions classified as responders. Further, health economics analyses demonstrated a cost savings of 39% for the stepped care model relative to in-person CBT-ERP. These initial findings hold promise for stepped-care models for moderate severity OCD. However, it is not yet clear how well-suited stepped care is for more severe and complex OCD, given that unnecessary delays in adequate dosing of CBT-ERP may result in higher illness-related costs and deterioration in functioning for CYP at the more severe end of the spectrum (Diefenbach & Tolin, 2013).

NordLOTS (Torp et al., 2015), the largest dissemination and implementation trial of CBT-ERP for paediatric OCD, involved a stepped care model of first-line, in-person CBT-ERP delivered across three Scandinavian countries (*n* = 269 youth), including 20 community mental health clinics. All CYP received an adequate dose of CBT-ERP, consisting of 14 weekly sessions as first-line care (Step 1), with 89.6% completing the full treatment. There were significant overall improvements in OCD symptoms from pre- to post-treatment with an overall 52.9% reduction in OCD symptoms. This multi-national, multi-site trial of evidence-based CBT-ERP for OCD has been a landmark trial for implementation of CBT-ERP in routine care at scale, providing evidence that CBT-ERP, following rigorous clinician training and supervision, can be implemented in community mental health settings with positive outcomes similar to those achieved in clinical trials.

In regard to non-responders, CYP who were assessed as having persistent OCD symptoms at 6 months follow-up (i.e. ≥ CY-BOCS 16) (following Step 1) were then randomised to either (a) continued CBT, or (b) SRI for additional 16 weeks (Step 2: Højgaard et al., 2017). Of the original 269 participants in the NordLOTS study, 27.4% (*n* = 66) were determined to be non-responders to CBT and eligible for Step 2 treatments. Results found large within-group effects sizes for both conditions, suggesting continued treatment for CBT non-responders is beneficial. Moreover, continued CBT-ERP for non-responders to first-line CBT resulted in significant benefits, with 50% meeting clinical response with further CBT-ERP, an effect that was not significantly different to augmenting first-line CBT with SSRI treatment of non-responders.

Notably, an additional analysis of the NordLOTS data, examined early response, at mid-treatment (week 7), to first-line weekly CBT-ERP. After only 7 sessions, 4 out of 10 CYP with OCD achieved early and persistent response to CBT-ERP, with a 50% mean reduction in symptoms, equivalent to below clinical threshold. The authors examined predictors of early response and found that youth with lower baseline OCD severity were more likely to achieve this rapid and clinically significant response to CBT-ERP.

Taken together, these compelling findings highlight the importance of optimal dosing of CBT-ERP, suggesting a sizable proportion (40%) of CYP with OCD can achieve a good clinical response with only 7–10 h CBT-ERP; whereas others (20–30%) may require a much stronger dose of CBT-ERP than is typically recommended. The finding that continued CBT-ERP for non-responders is an effective alternative to SRI augmentation has enormous clinical significance and is a finding that is contrary to current practice guidelines (Geller et al., 2012), which recommend routine SSRIs for non-responders to CBT; a recommendation that has not yet been empirically supported. In sum, achieving optimal patient outcomes from CBT-ERP for OCD may be a matter of dose. Innovative models of care that *personalise* CBT-ERP dose relative to clinical need (i.e. severity) may offer considerable efficiencies (i.e. time and cost) and reduce the treatment-quality gap.

## A New Direction: Staged-Care Models of Service Provision for OCD

A synthesis of the evidence for CBT-ERP across different modalities as well as clinical indicators of response (e.g. severity) has highlighted several important findings:

- CBT-ERP is widely effective for CYP with OCD, across all modalities, including in-person individual or group delivery, internet-delivered, and intensive and brief interventions.
- There are several, multi-level barriers to accessing high-quality care, including lack of specialised clinical training and supervision in CBT-ERP implementation.
- High-quality CBT-ERP can be implemented effectively, at scale, in routine mental health services with sufficient training and supported implementation.
- Efficiencies and effectiveness may be leveraged via more personalised dosing of CBT-ERP; ensuring intensity of care flexibly meets clinical needs.
- Several known predictors appear to be associated with poorer response to first-line CBT-ERP, with the most consistent variables being greater OCD severity, and specific comorbidity (e.g. depression, disruptive behavioural disorders, neurodevelopmental disorders—ADHD and ASD). These predictors could serve to inform whom may need less, or more, of empirically supported CBT-ERP.
- Intensively delivered CBT-ERP appears to be more robust against clinical characteristics that predictor poorer response to first-line CBT-ERP, such as comorbidity.

Staged care represents a major step forward in mental health care that aims to deliver highly personalised care, delivering “right care first time” (Sawrikar et al., 2021). As noted earlier, in staged care, patients receive hierarchically arranged service packages that vary according to the intensity, duration, and mix of treatment options, with provision of care from prevention, early intervention, through to first and second-line treatments. A range of clinical indicators is considered, where evidence exists, to inform clinical decision-making and stage of care, including symptom severity, duration, functioning, and previous treatment response determining the level of care using a single prognostic index (McGorry et al., 2006). Staged care represents an innovative population health-oriented service delivery model that incorporates clinical need in allocation-of-care decisions to personalise the treatment for individual patients.

Prior research demonstrates that patients in milder stages of illness respond better than those with severe illness to less intensive treatment approaches (Cross et al., 2016, 2017), which is also supported by evidence from CBT-ERP for OCD (Torp & Skarphedinsson, 2017). However, more intensive care is required for patients in later stages of illness (Salagre et al., 2018), which includes integrated approaches, alleviating current symptoms and risk, while targeting longer-term functional improvement to prevent further illness progression (Colizzi et al., 2020; Sawrikar et al., 2021).

Sawrikar et al. (2022) have recently described a clinical staging framework for youth at risk of developing internalising and externalising disorders. According to their model, the early stages are related to non-specific symptoms with presentation of risk factors (0), mild clinical symptoms with low risk of progression (Stage 1a) that then give way to sub-clinical syndromes that are more likely to precede to full-threshold syndromes (Stage 1b). Subsequent stages capture progressed illness full-threshold syndromes, characterised by clinically significant severity and impairment (stage 2) and then to progression to recurrent, persistent, and treatment-resistant forms of illness (stage 3 +). Staged-care frameworks such as this can be tailored for other mental health disorders, such as OCD, to improve treatment outcomes for CYP with sub-clinical symptoms through to full-threshold illness.

Fontenelle and Yücel (2019) recently proposed a four-stage care model for adult OCD treatment, whereby at Step 0, individuals with no symptoms (Y-BOCS = 0) and mild risk factors (e.g. family history of OCD) receive “watch and wait” treatment. Step 1 includes individuals with sub-threshold symptoms (Y-BOCS = 1–13) and multiple risk factors who would receive psychoeducation, group CBT, meditation, or exercise therapy. Those in Step 2 with moderate symptoms (Y-BOCS = 14–34) are recommended SRIs, ERP, and/or transcranial magnetic stimulation (TMS). For patients with the most severe/extreme OCD (Y-BOCS = 35–40), the authors recommended SSRIs, ERP, and/or TMS treatments, and, when treatment resistant, also considered deep brain stimulation (DBS) or psychiatric surgery. Based on our review of the literature, this is currently the only model of staged care proposed for CYP with OCD, and currently, there is no model of staged care for CBT-ERP specifically, in the context of children and young people with OCD. This staged care model for OCD, although not empirically validated nor vetted by the expert community, recognises that treatment outcomes for patients with OCD may be improved through more precise, personalised matching of treatments to the level of clinical need. This clinical framework may improve access to care and reduce demand for higher intensity interventions via early identification and more rapid delivery of care for those CYP with sub-threshold symptoms, whom also experience impaired functioning and distress. Given that OCD most frequently onsets at around 10 years of age (range 6–12 years) (Geller et al., 2021), and that early onset OCD is associated with greater severity, higher comorbidity, and a poorer prognosis and treatment response relative to adult onset OCD (Geller et al., 2021; Taylor, 2011), development of a staged-care model for CYP with OCD utilising evidence-based treatments, tailored specifically to the level of clinical need is warranted and may provide a bridge for the current treatment and quality gap in mental health services for young people.

## Closing the Gap: Novel Staged CBT-ERP for CYP with OCD

Based on cumulative evidence for the effectiveness of CBT-ERP for CYP with OCD, across in-person weekly individual and group delivery (Barrett et al., 2004; Farrell et al., 2010, 2012a; Lavell et al., 2016), brief and intensive formats (e.g. 2022b; Canavera et al., 2022; Farrell et al., 2016), and multi-technology modalities (e.g. Farrell et al., 2023; Scott et al., in preparation), we proposes a preliminary model of staged CBT-ERP for CYP with OCD that tailors level of care (i.e. CBT-ERP intensity) based on level of clinical need, informed by evidence on known determinants of CBT-ERP treatment response.

**Fig. 1:**
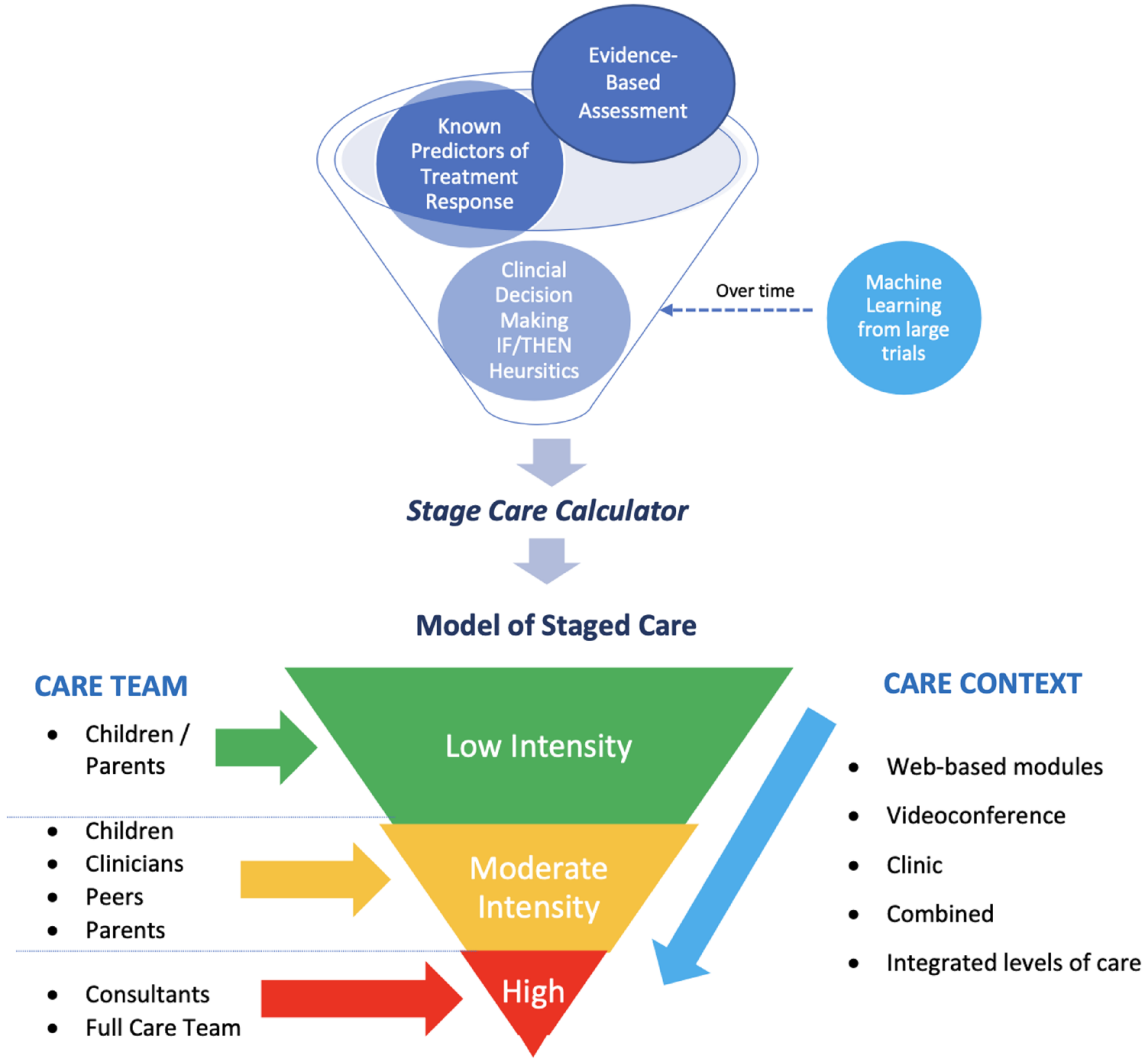
A conceptual framework to guide staged-care CBT-ERP for CYP with OCD

Moreover, our model personalises CBT-ERP by considering essential elements of the *care team* (i.e. clinicians, parents, peers, multidisciplinary consultants) and *care context* (i.e. digital, home, clinic) at each stage of the model to ensure *efficiencies* wherever possible, while maintaining adequate *intensity* and thus safeguarding *efficacy*. Figure 1 illustrates the conceptual framework that guides our model of staged-care CBT-ERP for OCD.

### Evidence-Informed Staging

Reliable screening is the first step in determining staged-care CBT-ERP for CYP with OCD. Although there are several reliable screening and assessment measures available for OCD in CYP (e.g. Children’s Florida Obsessive–Compulsive Inventory, (Storch et al., 2009), we recommend brief screening tools in the first instance to determine clinical need. For instance, there are two brief screeners available for OCD in CYP, including the Short OCD Screener (Uher et al., 2007) and the Obsessive–Compulsive Inventory- Child Version-5 (OCI-CV-5; Abramowitz et al., 2022). In relatively small clinical samples, the 7-item SOCS has been found to have good reliability and acceptable sensitivity and specificity for detecting OCD (Uher et al., 2007).

More recently, in a large clinical sample of CYP with OCD (*n* = 489), as well as non-clinical controls (*n* = 259) and youth with other mental health disorders (*n* = 299), Abramowitz et al. (2022) found the 5-item OCI-CV-5, that assesses different dimensions of OCD (washing, checking, ordering, obsessing, neutralising/counting), resulted in good to excellent psychometric properties, and a cut-off of (≥ 2) yielded optimal sensitivity and specificity. To detect early signs of OCD, including sub-threshold OCD, we would recommend a score of ≥ 1 to inform a full staged -care model; although, further research is needed to validate this screening tool for such purposes. Nevertheless, clinical guidelines for paediatric OCD recommend routine screening within mental health services (Geller et al., 2012).

Following detection of possible OCD, full clinical assessment is warranted to inform diagnosis and clinical severity of OCD, using a structured diagnostic interview (to determine comorbidity and differential diagnosis; e.g. Anxiety Disorders Interview Schedule for Children, Parent and Child versions (Silverman & Albano, 2022) along with the gold-standard CY-BOCS (Scahill et al., 1997). The CY-BOCS is comprised of two symptoms checklists (obsessions and compulsions) and five subscales (time, distress, interference, resistance, control) that indicate obsessions severity, compulsion severity, and total severity. It is administered in a semi-structured interview format, delivered to parents and children conjointly, either in-person, or via videoconference. The CY-BOCS has demonstrated good to excellent internal consistency (Cronbach's *α* > 0.94) (Abramowitz et al., 2022), interrater reliability, and test–retest reliability (Scahill et al., 1997). Recently, Cervin and colleagues developed empirically supported severity benchmarks for the CY-BOCS based on cumulative data from 5140 patients with OCD across 5 nations (Cervin et al., 2022). Based on this work, severity cut-offs to inform clinical staging are; sub-clinical OCD: 1–13 points; mild OCD: 14–21 points; moderate OCD: 22–29 points; severe OCD: 30–40 points. Overtime and following larger scale implementation, we expect the use of artificial intelligence and machine learning, using baseline “risk” data, combined with patient “outcome” data to result in algorithms that can inform refinement to the proposed clinical staging model over time.

We propose that clinical staging for paediatric OCD be informed by the past two decades of research from clinical trials and meta-analyses, which have informed a range of predictor variables associated with treatment response to evidence-based CBT-ERP for OCD. While there is no formal consensus on definitive markers for early, partial, or delayed response to CBT-ERP for OCD, there has been consistent evidence that baseline ***severity***, as indexed by the CY-BOCS (higher severity), is associated with attenuated response, for both early and acute post-treatment response to CBT-ERP (Garcia et al., 2010; Højgaard et al., 2020; Piacentini et al., 2002; Turner et al., 2018). While there is more variability across studies in which *comorbidity* has been found to predict poorer response, there is general consensus that comorbidity is a complicating factor in CBT-ERP response, particular for CYP with severe depression, ADHD, and/or ASD.

There are of course other complex comorbidities which may (and frequently do) result in poorer, or slower response, including comorbid eating disorders, psychosis, bipolar disorder, and Tourette’s Syndrome, which to date there is no empirical evidence since these CYP are often excluded from RCTs. However, we propose that all of these conditions be considered “complex comorbidity”, likely to be associated with poorer response to CBT-ERP, and thus inform higher entry into staged care. Thus, we propose a preliminary *staging algorithm* to determine level of clinical need, based on the current state of the empirically literature for CBT-ERP, utilising three key determinants:

- *Symptom level and severity*, as indexed by a positive OC screen (i.e. ≥ 1 on the OCI-CV-5), and OCD symptom severity based on empirically supported CY-BOCS cut-offs (sub-clinical 1–13; mild 14–21; moderate 22–29; severe 30–40).
- *Comorbidity,* as indexed by the presence of co-occurring complex mental health disorders, including (but not limited to) *severe* depression, disruptive behavioural disorders, AD/HD, and ASD, based on a structured diagnostic clinical interview (e.g. Anxiety Disorders Interview Schedule for DSM 5, Child and Parent versions, (Silverman & Albano, 2022); The Schedule for Affective Disorders and Schizophrenia for School-Age Children: Present and Lifetime version, (Kaufman & Schweder, 2004; Piacentini et al., 2002).
- *Treatment history,* based on current best-practice guidelines and good clinical care, we propose that a failed (and adequate) past trial of CBT-ERP for OCD also represents a level of increased clinical need.

**Fig. 2:**
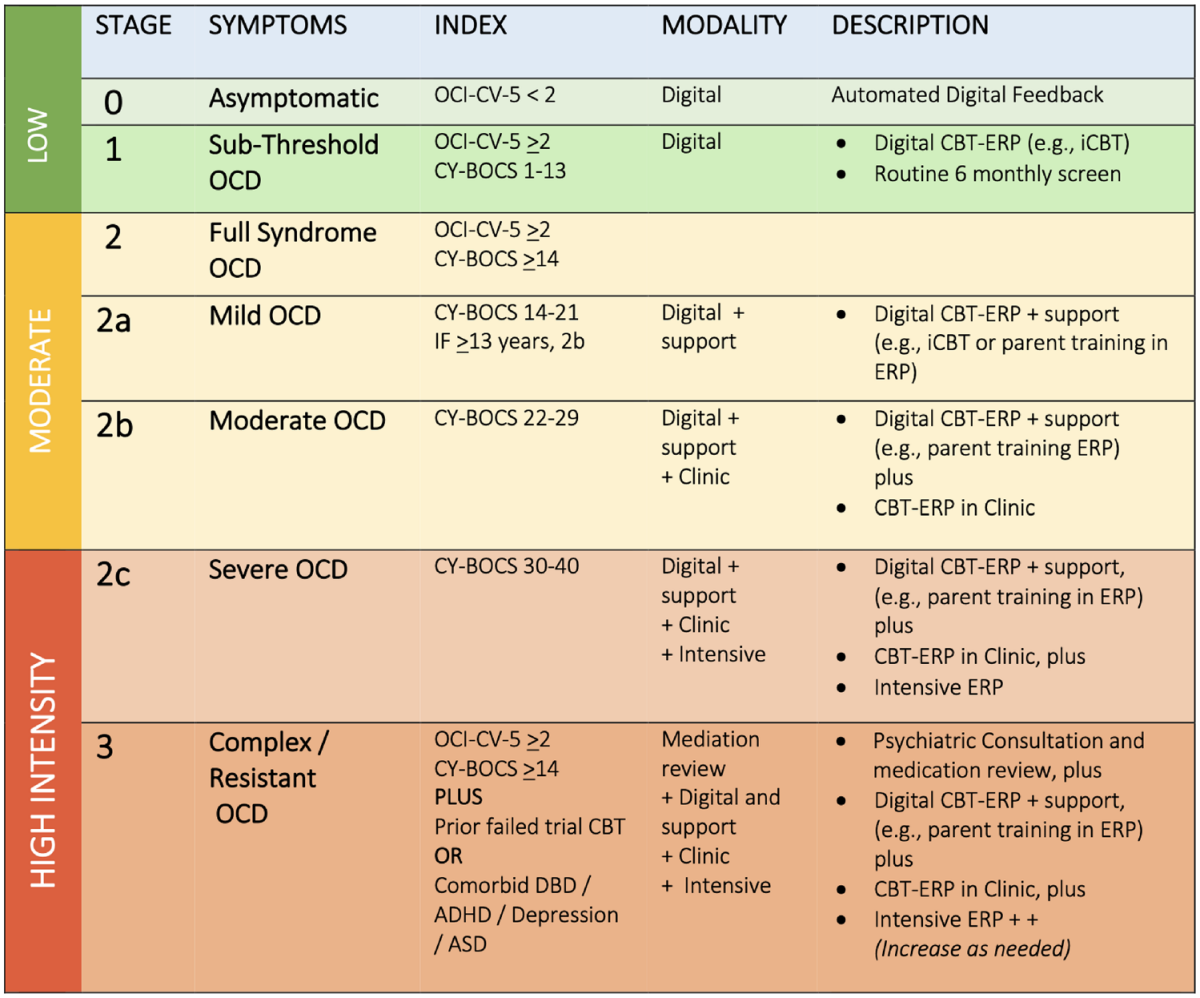
Staged CBT-ERP for CYP with OCD

Figure 2 illustrates our proposed model of staged-care CBT-ERP for CYP with OCD, which should guide clinicians in determining level of care using our recommended staging algorithm, based on known determinants of response, including OCD symptom severity, comorbidity and prior OCD treatment history. Notably, this staging model represents a model of care for CBT-ERP, with or without medication management. While we specifically indicate formal psychiatric review and possible medication management plan for complex and resistant OCD (stage 3), we do not preclude children and young people from being on medication at earlier stages. Instead, we recommend clinicians adhere to current evidence-based guidelines for management of OCD and recommend medication management at any of the earlier stages, where necessary to improve treatment outcomes for young people (e.g. for low insight/motivation to engage in CBT-ERP, or lack of treatment providers of CBT-ERP, (Geller et al., 2021). For CYP that are acutely unwell and/or actively suicidal, we recommend routine comprehensive clinical assessment, including risk assessments, and inpatient admissions when required. The proposed model of care relates to clinical services delivered in the community by appropriately trained clinicians in CBT-ERP for OCD (Piacentini et al., 2021; Sookman et al., 2021).

### Staged CBT-ERP for OCD in CYP

The model assumes that lower intensity care should be offered to all children and youth with sub-clinical to mild OCD, utilising models of treatment that affords greater scale, including digital interventions (i.e. versions of iCBT with minimal therapist support (e.g. Lenhard et al., 2017, 2020) that can efficiently reach more children. For CYP with moderate OCD and higher clinical need, we propose multi-level CBT-ERP, engaging a more *comprehensive care team* via inclusion of clinicians, parents, and peers (group CBT-ERP, which affords economy of scale in service provision), with implementation of ERP supported across different contexts, in the clinic and at home (i.e. via use of technology to support parents and children within the home, e.g. multi-modality parent training in CBT-ERP (Farrell et al., 2022a). For CYP with severe OCD, a multi-level approach that provides an even higher level of care via the addition of intensive delivery ERP to standard first-line CBT-ERP (or as a standalone intervention if sufficiently dosed), along with parent-focussed training/support is recommended (i.e. digital provision of parent training in CBT-ERP for OCD; Farrell et al., 2022a). Finally, for complex OCD, defined by the presence of specific “complex” comorbidity or treatment resistance, we propose fully coordinated and multidisciplinary care teams, that include psychiatry review and medication management, along with parent and family support, and multi-level implementation of CBT-ERP, including standard CBT-ERP, intensive delivery of ERP (dose as needed), and family support, across contexts where possible (clinic and ERP within the home via use of technology). An innovation in this model is that treatments are not linear—children do not have to progress from one modality to another, if the treatment is unsuccessful. Instead, patient outcomes are optimised by providing *mixed-modality*, concurrent delivery of CBT-ERP, using complementary interventions across care teams and care contexts.

Our model proposes incremental increases in dose of CBT-ERP relative to clinical need; a model which balances the need for treatment efficiencies in closing the access gap, along with the need for high-quality specialised CBT-ERP to close the quality gap. Essential to this model of care is high-quality, clinician training in specialised CBT-ERP for CYP with OCD, along with ongoing clinician supervision in implementation. As level of care increases in this model, so does the level of expertise required of the treating clinicians to flexibly deliver, personalised intensive ERP across contexts (in-person where possible and in-home utilising videoconference technology).

We propose three broad levels of care commensurate with clinical need, with more nuanced delivery of CBT within each of these three levels of intensity.

(1) *Low -intensity digital* CBT-ERP for sub-threshold OCD [Stage 1a: digital psychoeducation] to mild OCD [Stage 1b: therapist-assisted, digital CBT-ERP] before illness results in major impairment.

There are several therapist-assisted iCBT programs for OCD, including BIP for adolescents with OCD (aged 12–17 years) that includes 12 online modules (chapters) along with five online parent modules that supports (Lenhard et al., 2016) young people to overcome OCD (Aspvall et al., 2018). BIP has been tested in a pilot study (*n* = 21), as well as a randomised controlled trial (*n* = 67; Lenhard et al., 2017) and found to be effective at reducing OCD symptoms, and requires only minimal weekly therapist support (e.g. 17.5 min per week). The intervention includes modules on psychoeducation, exposure and response prevention, and cognitive interventions (Lenhard et al., 2016). Another iCBT program, includes “OCD? Not Me!” (Rees et al., 2016), a full automated iCBT program that delivers 8 stages of treatment aimed at teaching children (aged 12–18 years) how to overcome OCD. Modules include psychoeducation, exposure and response prevention, cognitive therapy, and dealing with stress and setbacks. Self-guided interventions might be particularly well suited for sub-threshold OCD, with preliminary evidence to support reduction in OC symptoms for youth with sub-threshold and clinical levels of OCD (Rees et al., 2016). More recently, we have developed a brief multi-technology parent training intervention of CBT-ERP for OCD (Farrell et al., 2022a; Scott et al., in prep). This intervention, based on OCD BUSTERS (Farrell & Waters, 2008) incorporates 4 web-based, self-directed weekly modules for parents, combined with 4 weekly videoconference group sessions to train parents in the “how” to implement and support ERP practice within the home. This modality of care focusses on equipping parents with long-lasting skills to support their child in overcoming OCD, where parents are and when they need it FAST (**F**amilies **A**ccessing online **S**kills **T**raining in ERP). FAST CBT includes modules on psychoeducation, training in implementing ERP with children, contingency management for ERP practice and reduction of family accommodation. This modality draws on “transfer of control theory” (Silverman & Kurtines, 1996), shifting the control / implementation of therapy from the therapist to the parent, increasing parental engagement and mastery in managing OCD and facilitating ERP practice, and reducing family accommodation, both established obstacles in child-focussed interventions.

(2) *Moderate-intensity mixed-modality* CBT-ERP for full syndrome, moderate severity OCD [Stage 2: individual or group CBT-ERP in clinic, plus therapist-assisted, digital parent training in ERP].

All children with clinical and moderate level severity of OCD should receive empirically supported, specialised, first-line clinic-based CBT-ERP (i.e. individual and/or group, weekly in-person CBT-ERP, typically 14 + sessions). We propose wherever possible group CBT-ERP (e.g. 2012a; Farrell et al., 2010; Lavell et al., 2016) be considered, since this modality has been demonstrated to be as effectives as individual CBT-ERP in RCTs (Barrett et al., 2004), yet offers efficiencies in allocation of care within busy mental health clinics. Moreover, group CBT-ERP harnesses the power of peers, providing opportunity for peer modelling of coping, ERP practice and success, positive peer reinforcement for practice and incremental success, thus providing enriched opportunities for learning, mastery and motivation. Given that family accommodation occurs at high rates among children with OCD, and that family accommodation is associated with more severe OCD and a poorer response to treatment (e.g. Gorenstein et al., 2015; Lebowitz et al., 2012), we recommend family-focused CBT-ERP intervention for CYP with moderate severity OCD and above. Family-focused CBT-ERP (e.g. Barrett et al., 2004; Freeman et al., 2014; Storch et al., 2007) involves the inclusion of parents in treatment, either for some component of every child session, or via additional parent-focused modules. In family-based CBT-ERP, parents are included as part of the child’s “expert team” and assist the child with at-home implementation of ERP, rewarding the child’s progress and reducing family accommodation. At this point in our staging model, we recommend our parent training in CBT-ERP, as an example, for the digital augmentation of clinic-based CBT-ERP, given the concentrated focus in this modality on parental support and reduction of family accommodation. Thus, at this stage in our model of care, we highlight the importance of care teams, including children, parents, clinicians, and peers to optimise child treatment outcomes.

(3) *High-intensity multiple modality* CBT-ERP for severe OCD [Stage 3a: group CBT-ERP, PLUS therapist-assisted, digital parent training, PLUS 1–3 individual, intensive ERP session/s); and unremitting or complex OCD [stage 3b: psychiatry review PLUS stage 3a and additional intensive ERP until treatment response achieved].

Individual, intensive ERP sessions can be delivered as a single session, or more, as needed, at any point during treatment. This modality provides a time efficient, concentrated dose of ERP, providing massed extinction learning over a prolonged session, speeding up gains of CBT-ERP in a “power dose” of within-session, therapist-assisted ERP. In high-intensity staged CBT-ERP, we propose the implementation of complimentary modalities of CBT-ERP providing unique care contexts, each of which we propose offers added benefits. Having a child participate in a weekly clinic-based group CBT-ERP program (~ 14 weeks), while parents engage in an online parent training program at home (4 weeks + brief maintenance support), combined with an intensive 3-h ERP session (or more) within the clinic or at home, provides a multi-level, optimised treatment that harnesses the support of parents, peers, and technology as unique, yet complimentary care contexts to robustly treat OCD in CYP with severe, complex, and/or refractory presentations.

## Evaluation and Implementation in Practice: Future Directions

Despite the well-documented distress and burden associated with childhood OCD and the availability of empirically supported treatments, there remains an unacceptable “treatment gap” and “quality gap” in the provision of services for CYP suffering from OCD. In response to a global crisis in mental health, marked by increasing demands for care within a sector of widening service gaps, recent innovations in mental health have argued for a staged-care approach to mental health service provision (Hickie et al., 2013; Ollendick et al., 2018; Sawrikar et al., 2021). A particular strength of this model is that it takes a risk-stratified approach to mental health service delivery, determining the level of care based on level of clinical risk/need, thus broadening the scope for intervening much earlier, incorporating prevention and early intervention potential into models of care (McGorry & Mei, 2018; Sawrikar et al., 2021). Based on two decades of empirical support for CBT-ERP for paediatric OCD since the first RCTs (Barrett et al., 2004; POTS, 2004), there is now considerable support for the efficacy of CBT via multiple modalities; a multi-nation trial of dissemination in routine community care (Torp et al., 2015); and international knowledge and competency standards under-pinning gold-standard implementation (Piacentini et al., 2021). Despite all of this—there remains an unacceptable treatment and quality gap for CYP with OCD.

Our review highlights that *dose* is an essential factor in determining the adequate response to CBT-ERP; with evidence to suggest a lower dose of CBT-ERP is likely to be sufficient for a substantive proportion of CYP with OCD (or sub-clinical OCD), while a much smaller proportion will require higher intensity care than typically delivered. Staged care presents a novel model of personalising CBT-ERP for CYP with OCD, by delivering the right level of care (i.e. dose of CBT-ERP), determined by clinical need (i.e. severity and complexity), first time. We have proposed a model of staged CBT-ERP for CYP with OCD that is evidence-informed and developmentally tailored to meet the personal needs of CYP with OCD and their families.

The future of staged-care CBT-ERP for OCD is dependent on the prediction accuracy of the staging algorithm, which is currently informed by a synthesis of empirical studies on predictors, several systematic reviews, and meta-analyses. To date, the evidence for predictors of response is mixed, and further studies with larger samples are required to improve our understanding of predictors of response to inform greater precision in staging algorithms. We propose a need for larger studies of implementation of CBT-ERP in community settings, including more diverse samples of young people with OCD than typically included in our RCTs that, combined with sophisticated machine learning statistical approaches, might unveil more nuanced prediction models of treatment outcomes. Machine learning is a powerful, statistical tool that can help sift through multi‐modal predictors and model their complex/non‐linear contributions, extracting sub-types of patients and their response to various treatment modalities (Chekroud et al., 2021). This work requires collaboration across teams and settings to work with large datasets to uncover more precise prediction models and move the field towards more personalised treatments for youth with OCD.

Relatedly, the current proposed staging model focuses on staging CBT-ERP, rather than the staging of multiple modalities of care, including pharmacological management of OCD. Indeed, a limitation of our proposed model is that it does not integrate biological treatment modalities at all, including pharmacotherapy, transcranial magnetic stimulation, and deep brain stimulation. The reason for this omission from our framework is a lack of evidence to inform staging of biological treatments for paediatric OCD, as well as associated risk and cost. Medications, although indicated for management of moderate-severe OCD (i.e. Geller et al., 2012), are associated with the risk of harm even if delivered with best practice. For instance, the first-line pharmacotherapy for OCD is SSRIs, which are associated with increased risk of osteoporosis (Perkes et al., 2016). Clomipramine is a second- or third-line medication in this age group; however, it is dangerous in overdose and associated with side effects (Fountain et al., 2020). Transcranial magnetic stimulation is expensive to access and has a far lower evidence base in this age group (Gregory et al., 2022); and finally, deep brain stimulation has an even lower evidence base and is also associated with serious risks (Rapinesi et al., 2019). For those reasons, we propose a model that supports implementation of CBT-ERP as a first-line treatment for all children and young people, with or without medication management; however, we specifically recommend psychiatry review of medication management for youth at the higher intensity spectrum of care.

In response to known barriers of implementation of CBT-ERP, and to enhance dissemination efforts, there is also a need for improved training and supervision of clinicians working with CYP with OCD in community mental health settings. Given that the availability of treatment manuals and clinical guidelines are insufficient in bridging the evidence-practice gap for CBT-ERP for CYP with OCD, international work has begun in developing training guidelines and ultimately standardised training protocols for the implementation of CBT-ERP (Sookman et al., 2021). A 14-nation International Obsessive–Compulsive Disorders Accreditation Task Force (ATF) of The Canadian Institute for Obsessive Compulsive Disorders (CIOCD) has been established to develop knowledge and competency standards for implementation of specialised OCD treatments (Sookman et al., 2021). Piacentini et al. (2021) published knowledge and competency standards for clinicians in delivery of specialised CBT-ERP for paediatric OCD based on a comprehensive literature review and expert synthesis, resulting in over 70 knowledge standards, and more than 60 specific competencies associated with evidence-based assessment and CBT-ERP treatment of CYP with OCD. The move towards established knowledge and competency standards (i.e. Piacentini et al, 2021), along with future work to develop a suite of accessible training protocols and resources, will hopefully serve to better equip clinicians with the knowledge and skills to utilise empirically supported CBT-ERP in routine clinical practice and support implementation of staged CBT-ERP.

Large implementation trials are needed of staged CBT-ERP for CYP with OCD to further inform and refine staging algorithms and determine the efficacy, cost-effectiveness, and long-term implementation potential in community settings. Future research and implementation would benefit from systematic co-design of each element within a model of staged care with mental health services; the use of published and empirically derived cut-offs for screening and assessment of paediatric OCD; assessment of an even broader range of clinical risk and prognostic indicators to inform refinements to staging algorithms (i.e. broader assessment of complex comorbidity, family history of OCD, past treatment history, etc.); and implementation within the context of rigorous clinician training and support to the levels of specialised, international standards in competency. The importance of flexibility of the model within routine care, with the overall objective to inform the delivery of high-quality care, within the context of resources available, is critical to successful implementation. One of the particularly appealing features of CBT-ERP is that it has been found to be effective across multiple modalities of care, including in-person, digital, and intensive formats. Access to high-quality and timely CBT-ERP can be facilitated by a model of staging, where lower intensity interventions of greatest reach can be accessed by all children and families regardless of the level of clinical need. Given the rise of telehealth services in delivery of routine care, we would recommend clinician-facilitated telehealth delivery of CBT-ERP for children who cannot access local in-person CBT-ERP. Government funding of OCD-specific treatment services and models of care (at least in Australia, Dyason et al., 2022) are needed to support the wider implementation of high-quality care for children and families struggling with OCD. In sum, though significant progress has been made in the treatment of youth with OCD, we cannot rest upon our laurels as much remains to be accomplished.
